# Editorial: Community series in biomarkers in the era of cancer immunotherapy: zooming in from periphery to tumor microenvironment, volume II

**DOI:** 10.3389/fimmu.2025.1662278

**Published:** 2025-08-01

**Authors:** Jehad Charo, Cristina Maccalli

**Affiliations:** ^1^ F. Hoffmann-La Roche AG, Pharmaceutical Research and Early Development Oncology, Roche Innovation Center Zurich, Zurich, Switzerland; ^2^ Unit of Biotherapies, Istituto di Ricovero a Carattere Scientifico (IRCCS) Ospedale Policlinico San Martino, Genova, Italy

**Keywords:** biomarkers, cancer, immunotherapy, statistic and modeling, fibroblasts, tumor microenvironment

Cancer immunotherapy (CIT) is firmly established as a standard of care in oncology, yet recent advancements have been staggered. To propel the field forward, it is crucial to learn from biomarker analysis. This learning is vital for two main reasons: identifying new targets or modalities and understanding the mechanisms of treatment response and resistance that influence clinical outcomes. This comprehensive approach would involve novel experimental wet-lab analysis, in-silico analysis, and sophisticated statistical techniques like machine learning or artificial intelligence (1–7).

This Research Topic of Frontiers in Immunology addresses these challenges, fostering a deeper understanding of recent developments in the field. We present 14 articles (10 original research and 4 reviews) that offer new insights into the role of biomarkers in patient enrichment, understanding response and resistance, identifying new targets, and comprehensively reviewing methods and approaches for CIT.

Dejardin et al. provide a comprehensive overview of statistical methods for analyzing early clinical trials in CIT, underscoring the vital role of biomarkers. They detail how biomarkers are used to understand the mechanism of action, optimize dosage, predict and manage adverse events, and identify patient subgroups for enrichment. This work serves as a single source for statistical models and principles for analyzing biomarker data, addressing challenges like high-dimensional data and correlating treatment-related changes with clinical outcomes to ensure robust and reproducible results. It also highlights the importance of validating biomarkers to establish their clinical utility.

Similarly, Qin et al. review biomarkers from diverse sources (tumor cells, microenvironment, liquid biopsy, gut microbiome, metabolites) and computational models (mechanistic and machine learning) for predicting the effectiveness of immune checkpoint inhibitor therapy in oncology. The article details detection methods, strengths, limitations, and applications, guiding researchers and clinicians in selecting appropriate tools for precision medicine.

Hou et al. reviewed the potential of circulating T cells as a promising biomarker for predicting and monitoring the effectiveness of anti-PD-(L)1 therapy in cancer patients. While traditional tumor-based biomarkers face limitations due to tumor heterogeneity, liquid biopsies, particularly analyzing different subsets of T cells from peripheral blood (memory, exhausted, effector T cells) offer a less invasive and potentially more representative approach. The authors discuss existing research on various T cell populations and their correlation with treatment outcome, highlighting both advantages and current limitations of using these biomarkers to inform therapy decisions.

Schlicher et al., extensively review the current status of small molecule inhibitors for CIT, focusing on a new class of drugs designed to overcome limitations of existing treatments. These molecules target intracellular negative regulators of anti-tumor immune responses, emphasizing mechanisms that either directly block suppressive feedback loops within immune cells or counteract immunosuppressive signals in the tumor microenvironment. Key targets covered include enzymes involved in T-cell receptor signaling (e.g. MAP4K1, DGKa/z, and CBL-B), phosphatases (e.g. PTPN2, PTPN22, and SHP-2), and modulators of adenosine and cGAS-STING pathways. The authors meticulously outline preclinical and early clinical trial data for numerous drug candidates, highlighting their potential in combination with existing immune checkpoint inhibitors and the ongoing efforts to identify predictive and pharmacodynamic biomarkers for patient selection and treatment efficacy.

Bargiela et al. explores how the factor inhibiting hypoxia-inducible factor (FIH), an enzyme that senses oxygen levels, regulates T cell function and their ability to fight tumors. The authors found that FIH’s effects on T cells, including their growth, differentiation, and cancer cell killing, depends on both hypoxia-inducible factor protein levels and oxygen concentration. Notably, removing FIH specifically in mouse T cells improved their effectiveness in CIT, suggesting that targeting this enzyme could be a promising strategy for treating cancer.

Papaevangelou et al. explored the synergistic effects of cytotopically modified interleukin-15, which allows IL-15 to anchor to cell membranes (cyto-IL-15), combined with the STING agonist ADU-S100, both administered directly into tumors. This combination effectively eliminated prostate tumors, prolonged survival, and conferred durable systemic immunity against tumor recurrence in a mouse model. The synergy was driven by activating both innate and adaptive immune responses, conferring resistance to subsequent tumor challenges.

Several studies in this Research Topic investigated biomarkers associated with resistance to checkpoint inhibitors and their potential utilization to enrich for responders or as candidates for standalone or combination therapies in CIT.

Nie et al. investigated Glycyl-tRNA synthetase 1 (GARS1) expression across multiple cancer indications, its prognostic value, and its relationship with the tumor immune microenvironment. GARS1 expression was found to be significantly upregulated in most indications (29 out of 33) compared to non-cancerous tissues and was associated with unfavorable survival. Through computational analyses and *in vitro* experiments on bladder cancer cells, the study identified GARS1 as a potential biomarker for predicting patient outcomes and informing therapeutic strategies effectiveness for GARS1-upregulated tumors.

Rodriguez et al. investigated a new strategy for overcoming resistance to anti-PD-1/CTLA-4 immunotherapy in lung cancer, a significant clinical challenge. While combination immunotherapy initially shows promise, tumors often develop acquired resistance, in part due to the accumulation of immunosuppressive cells, particularly Ly6C+ classical monocytes. The study demonstrates that targeting these Ly6C+ monocytes with an anti-Ly6C antibody can completely reverse this resistance, promoting the differentiation of these monocytes into anti-tumor dendritic cells and thereby reinvigorating the T-cell response for complete transplantable tumor eradication and control of autochthonous lung tumors growth.

Garman et al., performed comprehensive immunophenotyping of LAG-3 expression in tumor microenvironment (TME) and found that it is predominantly expressed by tumor-infiltrating CD8 memory T cells and frequently co-expressed with PD-1. These PD-1+ LAG-3+ CD8 memory T cells exhibit increased expression of activation and inhibitory markers, often accompanied by TOX, suggesting an exhausted state. In contrast, LAG-3 expression was more limited in circulating immune cells. The study also identified abundant expression of LAG-3 ligands, particularly MHC-II and galectin-3, across diverse tumor-infiltrating immune cells. Finally, elevated baseline and on-treatment levels of circulating LAG3 transcript-expressing CD8 memory T cells were associated with disease progression in melanoma patients treated with combination immune checkpoint inhibition. These insights support dual PD-1 and LAG-3 blockade.

Reis et al., investigated the role of tumor beta-2-microglobulin (B2M) and HLA-A protein expression in predicting responses to CIT. Using immunohistochemistry, the study found that the loss of these crucial proteins is a frequent event in various tumor types, particularly in metastatic cancer. A key finding is that immunotherapy can often reverse this protein loss, leading to increased B2M and HLA-A expression. While baseline levels of B2M or HLA-A alone were not definitive predictors of treatment success, their expression after treatment, particularly when analyzed in conjunction with other established biomarkers like CD8 and PD-L1, significantly improved the ability to predict positive responses to CIT.

Two studies investigated the role of TIGIT and its ligands in immunosuppression and their potential role as targets for CIT.

Liu et al., investigated the clinical significance of TIGIT ligand CD155 expression and its relationship with TME in gastric adenocarcinoma (GAC). Data from analyzing patient samples and public datasets demonstrated that increased CD155 expression correlates with GAC progression, poorer patient survival, and immunosuppressive CD68+ macrophages infiltrations. The authors suggest that CD155 could be a promising target for novel immunotherapies in GAC patients, given its role in disease progression.

Li et al., studied the immunosuppressive TME in hepatocellular carcinoma (HCC) using single-cell RNA sequencing (scRNA-seq). The authors investigated how various immune cells within the TME interact to promote tumor growth and specifically identified the TIGIT-PVR/PVRL2 axis as a key co-inhibitory signaling pathway, influencing the interaction between HCC cells, Treg cells, and exhausted CD8+ T cells. Tumor cell lysis and Granzyme B secretion significantly increased by blocking PVR or PVRL2 on HCC cells or TIGIT on immune cells. These findings propose the TIGIT-PVR/PVRL2 axis as a promising target for CIT in HCC.

Fibroblast Activation Protein (FAP) is both a prognostic marker and a potential target for CIT. Two articles in this Research Topic explored its prevalence and interaction with immune cells in the TME as well as its correlation with outcome in CIT.

Dziadek et al. investigated FAP as a biomarker for CIT across multiple tumor indications. They analyzed FAP expression and its correlation with patient outcomes in atezolizumab standalone or combination clinical trials. The study found FAP’s impact on therapy response to be complex, requiring more research to assess its value in predicting which patients would benefit from FAP-targeted treatments, particularly immunotherapy.

To further explore this, Kraxner et al. investigated FAP-expressing fibroblasts interaction in the TME and their relevance to CIT outcome. Immunohistochemistry and transcriptomics analyses showed that higher levels of FAP-positive fibroblasts correlate with increased T cell infiltration in some cancers, such as renal cell carcinoma, contradicting the notion that these fibroblasts exclude immune cells. These fibroblasts are associated with specific immune cells and cytokine signaling, indicating their influence on the TME and immunotherapy effectiveness. These findings underscore the significance of understanding the context of FAP-positive fibroblasts in the TME for designing more efficient personalized cancer treatments.

The published contributions have provided insights into the usage of non-invasive and invasive biomarkers from the TME, to determine the efficacy of a drug and inform on its optimal administration.

These valuable contributions aim to enhance our comprehension of CIT and to inform the development of future research. This, in turn, will facilitate more effective therapies and improved patient stratification in cancer treatment.
